# Multifunctional Gold Nanoparticles: A Novel Nanomaterial for Various Medical Applications and Biological Activities

**DOI:** 10.3389/fbioe.2020.00990

**Published:** 2020-08-13

**Authors:** Xiaopei Hu, Yuting Zhang, Tingting Ding, Jiang Liu, Hang Zhao

**Affiliations:** State Key Laboratory of Oral Diseases, National Clinical Research Center for Oral Diseases, Chinese Academy of Medical Sciences Research Unit of Oral Carcinogenesis and Management, West China Hospital of Stomatology, Sichuan University, Chengdu, China

**Keywords:** AuNPs, synthesis, modification, characterization, medical applications, biological activities

## Abstract

Nanotechnology has become a trending area in science and has made great advances with the development of functional, engineered nanoparticles. Various metal nanoparticles have been widely exploited for a wide range of medical applications. Among them, gold nanoparticles (AuNPs) are widely reported to guide an impressive resurgence and are highly remarkable. AuNPs, with their multiple, unique functional properties, and easy of synthesis, have attracted extensive attention. Their intrinsic features (optics, electronics, and physicochemical characteristics) can be altered by changing the characterization of the nanoparticles, such as shape, size and aspect ratio. They can be applied to a wide range of medical applications, including drug and gene delivery, photothermal therapy (PTT), photodynamic therapy (PDT) and radiation therapy (RT), diagnosis, X-ray imaging, computed tomography (CT) and other biological activities. However, to the best of our knowledge, there is no comprehensive review that summarized the applications of AuNPs in the medical field. Therefore, in this article we systematically review the methods of synthesis, the modification and characterization techniques of AuNPs, medical applications, and some biological activities of AuNPs, to provide a reference for future studies.

## Introduction

**FIGURE 1:**
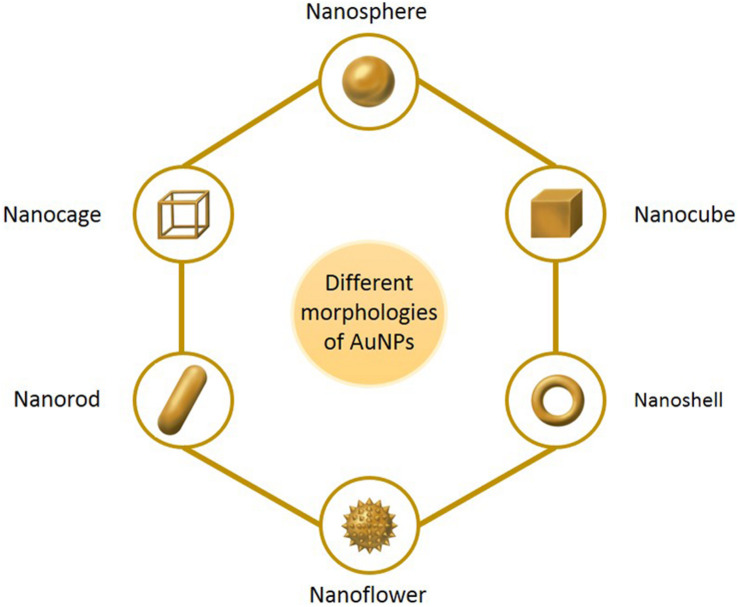
The main morphologies of AuNPs.

Nanomaterials are a novel type of material which has emerged in recent years. The term refers to a material in which at least one dimension, of three-dimensional space, is at the nanometer scale (0.1–100 nm), or is composed of the basic unit, which is approximately equivalent to the size of 10–100 atoms, is closely arranged together (Khan et al., 2017; Tayo, 2017). Nanoparticles are an example of nanomaterials, which now have the longest development time and are the most mature technology. Nanoparticles and nanotechnology are widely used and play an important role in a range of fields, such as medicine, biology, physics, chemistry and sensing, owing to their unique properties (Ramalingam, 2019). In comparison with other metal nanoparticles, noble metal (Cu, Hg, Ag, Pt, and Au) nanoparticles have increasingly attracted the attention of researchers (Ramalingam et al., 2014). Among these, gold nanoparticles (AuNPs) are known to be the most stable, and have now been prepared with various shapes and structures, including nanospheres, nanorods, nanocubes, nanobranches, nanobipyramids, nanoflowers, nanoshells, nanowires, and nanocages, by various synthetic techniques (Figure 1) (O’Neal et al., 2004; Chen et al., 2008; Li et al., 2015; Xiao et al., 2019). Moreover, they possess tunable and unique optical properties. Therefore, AuNPs have attracted extensive scientific and technological attention in recent decades. The optical properties of AuNPs are dependent on surface plasmon resonance (SPR), which is the fluctuation and interaction of electrons between negative and positive charges at the surface (Ramalingam, 2019). SPR can also be described in terms of surface plasmon polariton (SPP), which originates from propagating waves along a planar gold surface (Gurav et al., 2019). Due to their unique optical and electrical properties, and economic importance, AuNPs have abundant applications in various interdisciplinary branches of science, including medicine, material science, biology, chemistry and physics (Khan et al., 2019).

**FIGURE 2:**
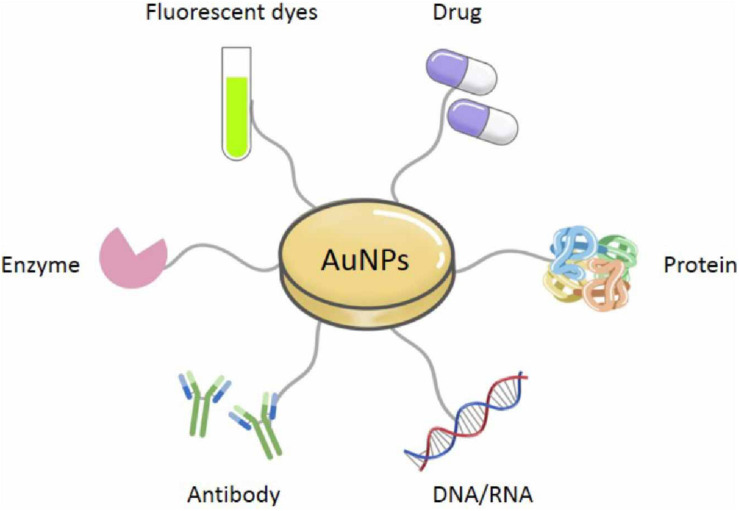
Various connecting molecules of AuNPs.

Especially, AuNPs are widely employed across the medical field owing to their excellent biocompatibility, which respectively results from their high chemical and physical stability, easy to functionalize with biologically active organic molecules or atoms (Pissuwan et al., 2019). AuNPs can directly conjugate and interact with diverse molecules containing proteins, drugs, antibodies, enzymes, nucleic acids (DNA or RNA), and fluorescent dyes on their surface, for diverse medical applications and biological activities (Figure 2) (Slocik et al., 2005; Ramalingam, 2019). Although AuNPs are so widespread and increasingly used in the medical field, there is no comprehensive review of their applications in medicine. Therefore, in this review, we have summarized the approaches that are available for synthesizing common AuNPs, as well as the techniques that are used to characterize them, based on their unique and diverse properties. We have also paid particular attention to the discussion of established medical applications of AuNPs.

## Synthesis and Modification of Multifunctional AuNPs

**FIGURE 3:**
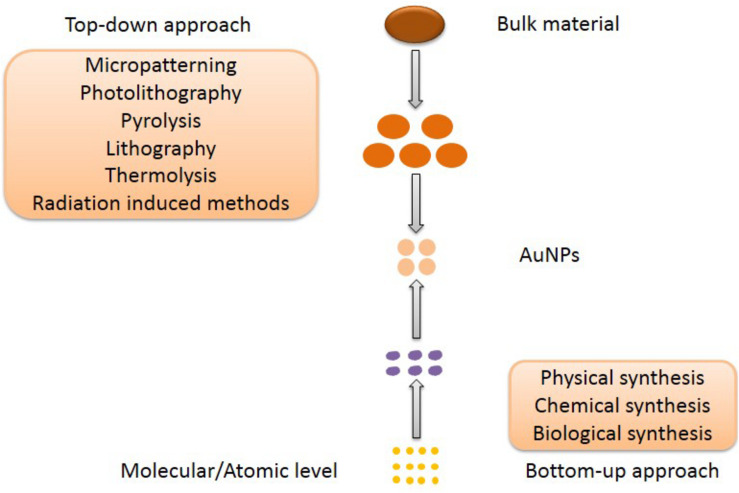
The top–down and bottom–up approaches for AuNPs synthesis.

Almost all the medical applications and biological activities of AuNPs was characterized based on the unique SPR, since the SPR can enhance the surface activity of AuNPs. Due to the excitation of SPR, the absorption spectrum connected with AuNPs shows a resonance band in the visible region, whose amplitude, spectral location and width can be modified by the diverse particle size and shape in the medium. Also, the SPR is strongly dependent on both size and shape (Ramalingam, 2019). Therefore, the preparation of size-controlled and shape-controlled AuNPs is essential for the medical applications and biological activities. The first report on AuNPs was published in 1857 by Faraday with light scattering potential of AuNPs confirmed by the change of red color and colloidal nature of nanomaterials (Faraday, 1857). Although AuNPs have a long history, the synthesis of small and stable structure of AuNPs is difficult, key challenge in nanotechnology. To our knowledge, there are two distinct approaches of synthesizing AuNPs, which are top–down and bottom–up respectively (Figure 3). The materials of AuNPs prepared by different methods are various, which are bulk material, small gold seeds or gold target, HAuCl_4_⋅4H_2_O and various biological extracts respectively. Furthermore, AuNPs can bind various active molecules, and have broad prospects in the application of diverse fields. Thus, the modification of AuNPs will also be introduced.

### Top–Down Approach

**FIGURE 4:**
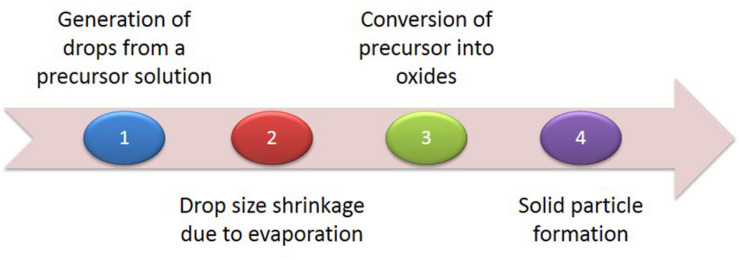
The four major steps of pyrolysis.

Generally, the top–down approach is a subtractive process, starting with the slicing of bulk materials and ending with self-assembled nanoscale objects (Khanna et al., 2019). Micropatterning and photolithography are the most common approaches (Chen et al., 2009; Walters and Parkin, 2009). Yun et al. (2006) demonstrated micropatterning of a single layer of nanoparticles and micelles through conventional and soft lithographical methods. Although the approach is fast, it has the limitation of synthesizing nanoparticles of uniform size. Thus, Chen et al. (2009) developed a novel patterning technique for AuNPs by removing salt-loaded micelles from substrate areas with a polymer stamp. They called the technique μ-contact (microcontact) deprinting, providing a fast and cheap way to produce nanoparticles on a wide range of substrates. In addition, there are several physical methods, such as pyrolysis, lithography, thermolysis and radiation induced methods in this category. Pyrolysis is another important technique frequently used, generally for the production of noble metal nanoparticles. As shown in Figure 4, pyrolysis has four major steps, from generation of drops from a precursor solution to solid particle formation (Figure 4) (Li et al., 2004). Pyrolysis has several disadvantages, such as the formation of porous films, low purity in some cases and limited products (Garza et al., 2010). In conclusion, the top–down approach has major limitations in the control of surface and structure of the AuNPs, which has a significant effect on their physical and chemical properties (Amblard et al., 2002; Sant et al., 2012). Size distribution is uncontrolled and enormous energy is required to maintain conditions of high-pressure and high-temperature during these synthetic procedures. Thus, it is very uneconomical and difficult to meet product requirements.

### Bottom–Up Approach

**FIGURE 5:**
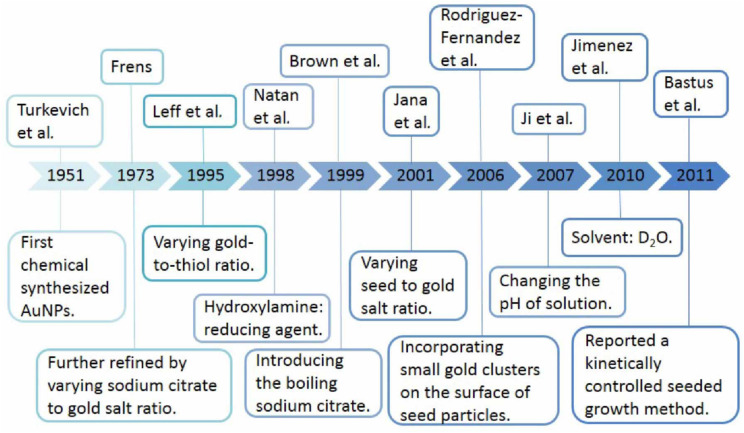
The chemical synthesis of AuNPs using different reaction conditions.

**FIGURE 6:**
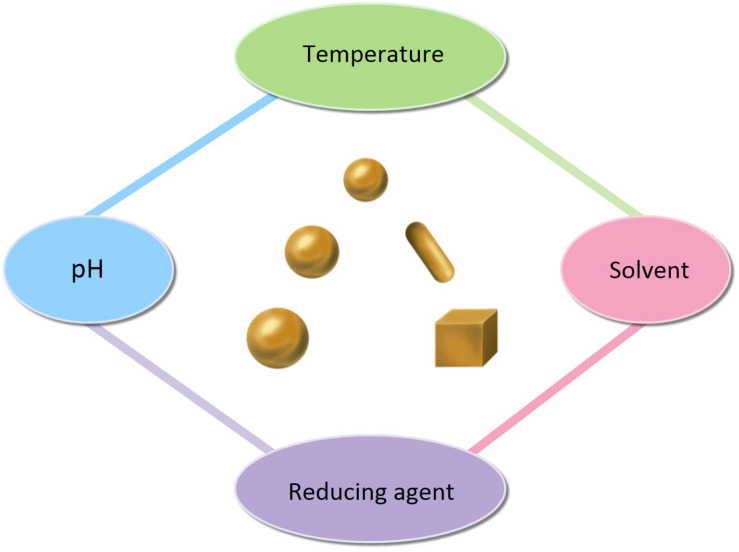
The factors affecting the size and shape of AuNPs.

As a popular nanomaterial, AuNPs are expected to present with applications in many areas. However, their yield is currently too low in existing methods of synthesis. Developing more convenient and adjustable methods to improve their preparation efficiency, in order to achieve production on a technical scale, has become the focus of research. The bottom–up approach has been an emerging strategy in recent years. There are three types of bottom–up synthesis approaches: (1) physical approaches, such as laser ablation, sputter deposition, ion implantation, γ-irradiation, optical lithography, microwave (MW) irradiation, ultrasound (US) irradiation, and ultraviolet (UV) irradiation (Table 1); (2) the chemical reduction of metal ions in solutions by introducing chemical agents and stabilizing agents, such as sodium hydroxide (NaOH), sodium borohydride (NaBH_4_), cetyl-trimethylammonium bromide (CTAB), lithium aluminum hydride (LiAlH_4_), sodium dodecyl sulfate (SDS), ethylene glycol (EG), and sodium citrate (Figures 5, 6); (3) biological approaches, using intracellular or extracellular extracts of prokaryotic cells (bacteria and actinomycetes) or eukaryotic cells (algae, fungi, and yeast), and extracts from various plants (leaves, stem, flower, fruits, peel, bark, and root) (Table 2). These syntheses will be discussed in detail in the following parts.

**TABLE 1:** Physical synthesis of AuNPs with different morphology and size.

Method	Morphology	Size (nm)	Author	References
γ-irradiation	Nanosphere	3–6	Le et al.	Le et al., 2019
Ion implantation	Crystalline	1.5–5	Morita et al.	Morita et al., 2017
Laser ablation	Nanosphere	10–15	Vinod et al.	Vinod et al., 2017
	Nanosphere	7	Hampp et al.	Riedel et al., 2020
Ultrasound irradiation	Polyhedral	15–40	Shaheen et al.	Bhosale et al., 2017
Microwave irradiation	Nanosphere	10–50	Luo et al.	Luo et al., 2018

**TABLE 2:** Organisms mediated synthesis of AuNPs with different morphology and size.

Organism	Morphology	Size (nm)	Author	References
*Garcinia mangostana*	Nanosphere	20–40	Nishanthi et al.	Nishanthi et al., 2019
*Couroupita guianensis*	Nanocube	15–37	Singh et al.	Singh et al., 2016a
*Acanthopanax sessiliflorus*	Nanoflower	30–60	Ahn et al.	Ahn et al., 2017
*Sporosarcina koreensis*	Nanosphere	92	Singh et al.	Singh et al., 2016b
*Sargassum swartzii*	Nanosphere	35	Prema et al.	Prema et al., 2015

#### Physical Approach

Most of the physical methods used to prepare nanoparticles involve controlling experimental parameters in the presence of a reducing agent, to modulate the structures and properties of AuNPs without contamination (Table 1). Laser ablation and ion implantation are the most common and important physical methods of synthesis. Laser ablation provides an approach which effectively alters the surface area, geometric shape, properties, fragmentation, and assembly of AuNPs in aqueous solution, a biocompatible medium (Correard et al., 2014; González-Rubio et al., 2016). For example, Vinod et al. (2017) synthesized pure AuNPs through laser ablation of a gold target in water, and these nanoparticles are inherently non-toxic. And these particles are photothermally active when excited with 532 nm laser irradiation. However, the yield of this method is low, and the method is inconvenient. Therefore, the development of convenient, high-efficiency methods is necessary, in order to scale up production. Recently, Riedel et al. (2020) synthesized spherical, silica-coated AuNPs, with an average diameter of 9 nm and a coating thickness of 2 nm, by improved pulsed laser ablation in liquid (PLAL), and this method offers great progress to the large-scale production of nanoparticles. Another promising method for synthesis of AuNPs is ion implantation, which has been extensively used to prepare AuNPs with precise physical, chemical, and biological properties. Nie et al. (2018) reported the synthesis of embedded AuNPs in Nd:YAG single crystals, using ion implantation, and subsequent thermal annealing. Both linear and non-linear absorption of the Nd:YAG crystals have been significantly enhanced.

#### Chemical Approach

The easiest and most commonly used approach to synthesis is the chemical reduction of metal ions in solutions (Figure 5). A typical synthesis of AuNPs is dependent on the reduction of Au(III) (from hydrogen tetrachloroaurate hydrate, HAuCl_4_) to Au(0) atoms, formed as clusters and accumulated into large, polycrystalline particles via aggregation in the presence of reducing or stabilizing agent. Citrate-stabilized AuNPs were initially synthesized by Turkevich et al. (1951), which was also the first chemical synthesis of AuNPs. This synthesis was based on the single-phase aqueous reduction of HAuCl_4_ by sodium citrate. This synthesis was further refined by Frens (1973) by varying the ratio of sodium citrate and gold salt in order to control the size of AuNPs, from 5 to 150 nm. However, the diameter (<30 nm) of AuNPs was too poor. Leff et al. (1995) synthesized surfactant-mediated AuNPs over a range of diameters from 1.5 to 20 nm, by varying the gold-to-thiol ratio (Leff et al., 1995). In 2007, adopting the classical reaction system, Ji et al. (2007) also synthesized AuNPs by changing the pH of solution, which can affect the composition of gold solute complexes, in order to alter the particle size. Then, Jimenez et al. (2010) synthesized small AuNPs with sodium citrate and heavy water (D_2_O). This was a faster reduction method, and by increasingly replacing water with deuterium oxide, smaller diameters were obtained. Today, the aqueous method remains the most commonly used. However, the shape of AuNPs is irregular, and the size and size distribution obtained are quite poor. Thus, Natan and Brown (1998) reported the seeded growth of AuNPs (up to 100 nm in diameter) by using hydroxylamine as a mild reducing agent. And Brown et al. (1999) prepared AuNPs with highly uniform shape and size by introducing the boiling solution of sodium citrate. The mean diameters of the AuNPs produced were between 20 and 100 nm, and they exhibit improved monodispersity. A similar procedure, utilizing the reductant NH_2_OH at room temperature, produces two populations of particles. The larger population is even more spherical than citrate-reduced particles of similar size, while the smaller population is very distinctly rod shaped. This work was improved by Jana et al. (2001) and Rodriguez-Fernandez et al. (2006). They synthesized monodispersed AuNPs with narrow size distributions, using ascorbic acid (AA) and CTAB, which are used as a reducing agent and cationic surfactant respectively. Jana et al. (2001) prepared the AuNPs with diameters of 5–40 nm by varying the ratio of seed to gold salt, whereas Rodriguez-Fernandez et al. (2006) prepared the AuNPs with diameters from 12 to 180 nm by incorporating small gold clusters on the surface of seed particles (Jana et al., 2001; Rodriguez-Fernandez et al., 2006). Although CTAB-based method can control the morphology of AuNPs, the thiolated cationic surfactant molecules that bind to the gold surface are difficult to remove and restrict further functionalization. The reason is that the strongly bound capping layer provided by the CTAB is difficult to exchange with the thiolated cationic surfactant molecules (Leonov et al., 2008). Thus, Bastus et al. (2011) reported a kinetically controlled seeded growth method for the synthesis of monodispersed citrate-stabilized AuNPs, with a uniform quasi-spherical shape of up to ∼200 nm, via the reduction of HAuCl_4_ by sodium citrate. They also evaluated the effect of temperature and pH on their final shape. According to the mentioned above, it is known that the temperature, pH, the solvent, and the reducing/stabilizing agent of the reaction system play a crucial role in controlling the size and shape of AuNPs (Figure 6). This has also encouraged researchers to look for novel strategies to prepare AuNPs with controllable properties. Recent seed-mediated synthesis methods are considered very efficient, with respect to precise control of the size and shape of AuNPs.

#### Biological Approach

Although the synthesis of AuNPs by physical and chemical methods gives a high yield and is relatively cheap, there are a few disadvantages which have also been reported, such as the use of carcinogenic solvents, the contamination of precursors, and high toxicity (Ramalingam, 2019). To overcome these difficulties, researchers have investigated the biological production of AuNPs, and have explored the potential of micro-organisms, due to the quest for economically as well as environmentally benign methods (Table 2) (Jain N. et al., 2011; Ramalingam et al., 2019). Biological systems and agents are excellent examples of hierarchical organization of atoms or molecules and this has caused researchers to use a wide range of biological agents as potential cell factories for the production of nanomaterials (Gardea-Torresdey et al., 1999; Singaravelu et al., 2007; Kasthuri et al., 2008; Smitha et al., 2009). Using biological agents to reduce the metal ions requires benign conditions of external temperature and pressure, and little organic solvent (Khan et al., 2019). For example, Dubey et al. (2010) reported a rapid, green synthesis for AuNPs, using the lower amounts extract of *Rosa rugosa* leaf (Kumar et al., 2010). They also evaluated the effect of the quantity of leaf extract, the concentration of gold solution, the stability of AuNPs and different pH with zeta potentiometer. Although environmentally friendly and easy to regulate the shape and size of the nanoparticles, bacterial-mediated synthesis also has disadvantages, such as difficulty in handling and low yield (Azharuddin et al., 2019).

### Modification

The size and morphology controlled AuNPs can be prepared based on different approaches above mentioned. AuNPs exhibit excellent physiochemical properties like unique SPR property, wide surface chemistry, high binding affinity, good biocompatibility, enhanced solubility, tunable functionalities for targeted delivery (Dreaden et al., 2012). Therefore, they have the ability to bind thiol and amine groups, which allows their modification for medical applications and biological activities (Shukla et al., 2005). On the one hand, AuNPs can directly attach ligands such as drug (Table 3), protein, DNA/RNA, enzyme, and so on (Figure 2). For instance, Podsiadlo et al. (2008) synthesized AuNPs bearing 6-Mercaptopurine (6-MP) and its riboside derivatives (6-Mercaptopurine-9-β-D-Ribofuranoside, 6-MPR). 6-MP and 6-MPR are loaded on the surfaces of AuNPs through sulfur-gold (Au–S) bonds known for their strength. They found substantial enhancement of the antiproliferative effect against K-562 leukemia cells compared to the free form of same drug. On the other hand, AuNPs are also used to conjugate with various drug with polymer functionalized for medical applications and biological activities. Recently, the design and preparation of polymer-functionalized AuNPs have attracted increasing interest. The AuNPs functionalized with polymer have more biocompatibility, stability, controlled release of drug, and enhanced therapeutic applications (Ramalingam, 2019). Some examples of polymer functionalized AuNPs for drug delivery are as shown in Table 3. For example, Venkatesan et al. (2013) developed AuNRs–doxorubicin conjugates (DOX@PSS-AuNRs) by an electrostatic interaction between the amine group (−NH_2_) of DOX and the negatively charged PSS-AuNRs surface. DOX@PSS-AuNRs conjugates exhibited improved drug loading efficiency, higher biological stability and higher therapeutic efficiency than free DOX. Therefore, the unique physical and chemical properties of AuNPs functionalized with/without polymer can enhance the efficiency of drug deliver and therapeutic efficiency, and increase the multifunctional application.

**TABLE 3:** Functionalized AuNPs without/with polymer for drug delivery with different morphology and size.

Polymer	Drug	Morphology	Size (nm)	References
–	6-Mercaptopurine	Nanosphere	4–5	Podsiadlo et al., 2008
–	Dodecylcysteine	Nanosphere	3–6	Azzam and Morsy, 2008
–	Kahalalide F	Nanosphere	20, 40	Hosta et al., 2009
–	Phthalocyanine	Nanosphere	2–4	Wieder et al., 2006
–	Rose Bengal	Nanorod	–	Wang et al., 2014
PEG	Doxorubicin	Nanosphere	11	Asadishad et al., 2010
PSS	Doxorubicin	Nanorod	5	Venkatesan et al., 2013
Chitosan	5-fluorouracil	Nanosphere	20	Chandran and Sandhyarani, 2014
Glycyrrhizin	Lamivudine	Nanosphere	16	Borker et al., 2016
PCPP	Camptothecin	Nanosphere	25–30	Sivaraj et al., 2018

## Characterization of Multifunctional AuNPs

**FIGURE 7:**
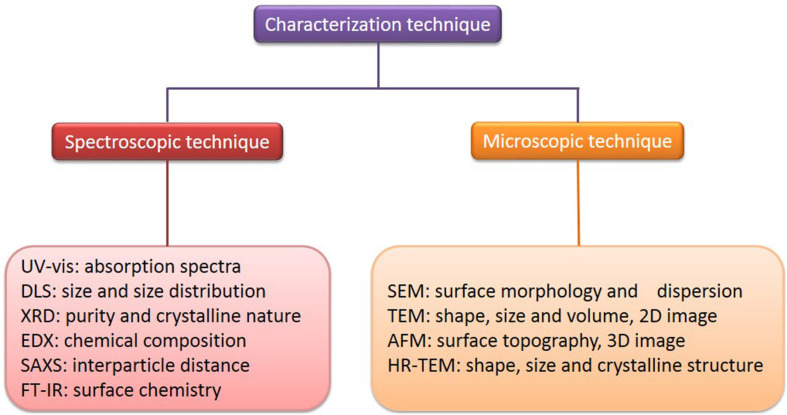
Characterization of AuNPs.

Various analytical techniques have been developed, in recent years, to characterize noble metal nanoparticles, according to their unique thermal, electrical, chemical, and optical properties, and to confirm their size (average particle diameter), shape, distribution, surface morphology, surface charge, and surface area (Roduner, 2006; Ray et al., 2015; Khanna et al., 2019). The characterization of AuNPs starts with a visual color change which can be observed with the naked eye, based on the principle of their unique and tunable SPR band (Ramalingam, 2019). The characterization of AuNPs has been shown schematically in Figure 7.

There are some indirect methods (spectroscopic technique) used to analyze the composition, structure, and crystal phase of AuNPs. Their striking optical properties are due to their SPR, which is monitored by UV-visible spectroscopy (UV-vis) (Sharma et al., 2016). The absorption spectra of AuNPs fall in the range of 500–550 nm (Poinern, 2014). It has been suggested a broadening of the SPR band width, which illustrates a redshift, can be used as an index of their state of aggregation, dispersity, size, and shape (Govindaraju et al., 2008; Shukla and Iravani, 2017). The size of AuNPs and their size distribution *in situ*, in the same range of hydrodynamic diameter, can be observed and measured by dynamic light scattering (DLS) (Wu et al., 2018). The purity and crystalline nature of AuNPs can be confirmed through X-ray diffraction (XRD), which gives a rough idea of the particle size, determined by the Debye-Scherer equation (Ullah et al., 2017). The chemical composition of AuNPs can be confirmed by energy-dispersive X-ray spectroscopy (EDX) (Shah et al., 2015). Small-angle X-ray scattering (SAXS) analysis can be used to provide a measure of the interparticle distance of AuNPs, of application to tumor imaging and tissue engineering (Allec et al., 2015). Fourier transform infrared spectroscopy (FT-IR) can investigate the surface chemistry to determine the functional atoms or groups bound to the surface of AuNPs (Dahoumane et al., 2016). The morphology of AuNPs can now be better characterized, due to recent developments in advanced microscopic techniques. These include scanning electron microscopy (SEM), transmission electron microscopy (TEM), high-resolution transmission electron microscopy (HR-TEM), and atomic force microscopy (AFM), which are commonly employed to determine and characterize their size, shape, and surface morphology (Azharuddin et al., 2019; Khanna et al., 2019). SEM provides nanoscale information about particles and determines their surface morphology and dispersion, while TEM is used to provide information about the number of material layers and broad evidence of uptake and localization, composition, polymer tethering, and physical properties (Marquis et al., 2009; Khanna et al., 2019). Also, TEM is commonly used as a quantitative method to measure size, volume, and shape, and it produces mainly two-dimensional (2D) image of three-dimensional (3D) nanoparticles (Quester et al., 2013). HR-TEM is used to determine the exact shape, size, and crystalline structure (Khanna et al., 2019). AFM, which is similar to the scanning probe microscopy, provides information about surface topography of AuNPs (Lu et al., 2004). AFM has the advantage of obtaining 3D images in a liquid environment (Lu et al., 2004; Khan et al., 2017). Some examples of the characterization of AuNPs, its morphology and size are as shown in Table 4.

**TABLE 4:** Characterization of AuNPs and its morphology and size.

Author	Morphology	Size (nm)	Characterization	References
Falagan-Lotsch et al.	Nanorod	16–50	TEM, DLS, UV-vis	Falagan-Lotsch et al., 2016
Dam et al.	Nanostar	40	TEM, DLS	Dam et al., 2014
Balfourier et al.	Nanosphere	4–22	TEM, STEM, HR-TEM, EDX	Balfourier et al., 2019
Ni et al.	Nanosphere	5, 13, 45	DLS, UV-vis	Ni et al., 2019
Lin et al.	Nanosphere	∼10	TEM, SEM, DLS	Lin et al., 2019
Dash et al.	Nanosphere	15–23	HR-TEM, UV-vis, EDX, XRD, AFM, FT-IR	Dash et al., 2014
Lee et al.	Nanosphere Nanooctahedra Nanocube	75	TEM, SEM, UV-vis	Lee et al., 2019

## Medical Applications of Multifunctional AuNPs

**FIGURE 8:**
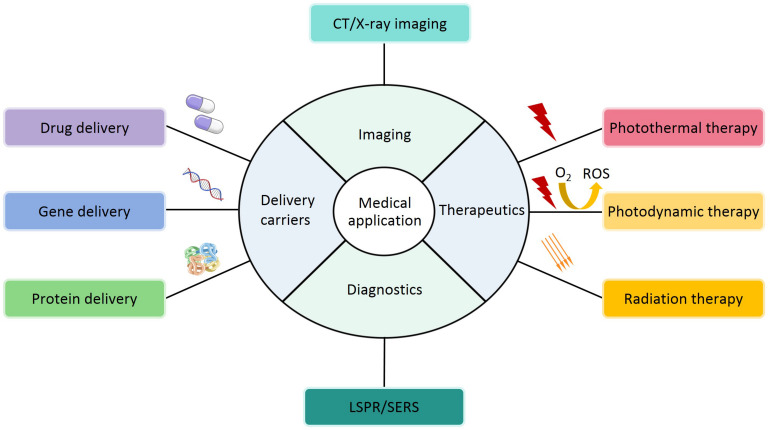
A schematic representation of medical applications for AuNPs.

In the above parts, the synthesis, modification and characterization of AuNPs based on optical and physicochemical properties have been introduced. Although nearly all studies are in the experimental stages, it is clear that AuNPs have potential applications in different fields. Based on their characteristics, applications have been explored, particularly in medical field, including deliver carriers (drug, gene and protein deliver), therapeutics (PTT, PDT and RT), diagnostics, imaging, and other biological activities (Figure 8 and Table 5). In the following sections, these applications will be discussed in detail.

**TABLE 5:** The application or activity of AuNPs with different morphology and size.

Author	Morphology	Size (nm)	Application/Activity	References
Tian et al.	Nanostar	40	PTT and CT	Tian et al., 2017
Rossi et al.	Nanosphere	5–10	Drug delivery and bioactivity	Rossi et al., 2016
Xu et al.	Nanocapsule	50	PTT, PDT and RT	Xu et al., 2019
Borkowska et al.	Nanocore	5.3 ± 0.7	Anticancer activity	Borkowska et al., 2020
Zheng et al.	Nanostar	7–10	PTT	Zheng et al., 2020
Liu et al.	Nanocapsule	30–40	Imaging	Liu et al., 2018
Venditti	Nanosphere	5	CT	Venditti, 2017
Yang et al.	Nanocube	50	PDT	Yang et al., 2018
Hu et al.	Nanosphere	100	PTT and RT	Hu et al., 2017
Yu et al.	Nanosphere	73.8	CT imaging and shRNA delivery	Yu et al., 2019
Zheng et al.	Nanosphere	2.04 ± 0.18	Drug delivery	Zheng et al., 2019
Shahbazi et al.	Nanosphere	19	Gene delivery	Shahbazi et al., 2019
Loynachan et al.	Nanocluster	2	Disease detection	Loynachan et al., 2019
Philip et al.	Nanosphere	37	SERS	Philip et al., 2018
Ramalingam et al.	Nanosphere	20–37	Anticancer and antimicrobial activity	Ramalingam et al., 2017
Filip et al.	Nanosphere	31	Anti-inflammation activity	Filip et al., 2019
Wang et al.	Nanobipyramid	–	Diagnosis	Wang et al., 2020
Ahmad et al.	Nanosphere	4–10	Antimicrobial activity	Ahmad et al., 2013
Tahir et al.	Nanosphere	2–10	Antioxidant activity	Tahir et al., 2015
Terentyuk et al.	Nanosphere	62	Antifungal activity	Terentyuk et al., 2014
El-Husseini et al.	Nanosphere	15	Diagnosis	El-Husseini et al., 2016

### Delivery Carriers

**FIGURE 9:**
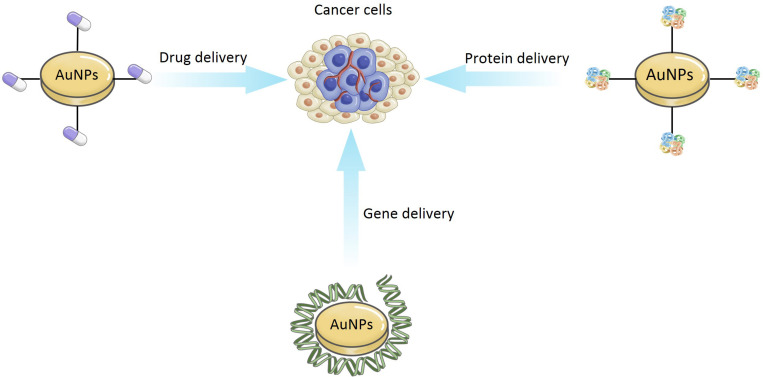
The application of delivery carriers for AuNPs.

In recent years, the idea of using AuNPs as delivery carriers has attracted the wide attention of researchers. As shown in Figure 9, AuNPs can be used for the delivery of drug, gene, and protein.

Chemotherapy is the most common method of cancer therapy but its potential is limited in many cases. Traditional drug delivery (oral or intravenous administration) for chemotherapeutic drugs, results in the dissemination of the drug throughout the whole body, with only a fraction of the dose reaching the tumor site (Singh et al., 2018). Targeting of specific cells, organs, and tissues, in a controlled manner, has become a key issue and challenge. Drug delivery systems (DDSs) is a promising approach to general anticancer therapy, which may provide efficient targeted transport and overcome the limitation of biochemical barriers in the body, e.g., the brain blood barrier (Martinho et al., 2011). Moreover, DDSs can enable controlled function in delivering drugs for early detection of the diseases and damaged sites (Baek et al., 2016). There are many useful forms for drug delivery, including liposomes, liquid crystals, dendrimers, polymers, hydrogels, and nanoparticles (Yokoyama, 2014; Rigon et al., 2015). Among these, only a small number of polymers and liposomes have been clinically approved (Piktel et al., 2016). Thus, many researchers have started to focus on the popular AuNPs. AuNPs have been examined for potential anticancer drug delivery (Duncan et al., 2010). In addition, they also can be easily modified to transfer various drugs, which may be bound to AuNPs through physical encapsulation or by chemical (covalent or non-covalent) bonding. Conjugation of AuNPs with other drugs is also possible, but it should be remembered that functionalization can change the toxicity of AuNPs, and their ability to successfully load or attach the desired drugs. The use of modified AuNPs has reduced systemic drug toxicity and helped to decrease the possibility of the cancer developing drug resistance (Yokoyama, 2014). For example, Wójcik et al. (2015) using the MTT (3-(4,5-dimethylthiazol-2-yl)-2,5-diphenyltetrazolium bromide) assay, confirmed that glutathione-stabilized AuNPs (GSH-AuNPs) modified with non-covalent conjugation of the DOX were more active against feline fibrosarcoma cell lines than the activity exhibited by unmodified AuNPs.

Gene therapy is the use of exogenous DNA or RNA to treat or prevent diseases. Viral vectors are commonly used but cannot be functionalized and can activate host immune systems (Riley and Vermerris, 2017). Their ‘design’ is inflexible, they target specific sites in a biological system with high cytotoxicity and reduce the efficiency of gene therapy (Riley and Vermerris, 2017). The use of non-viral vectors system (such as metallic nanoparticles) can solve this problem. Recent studies have shown that AuNPs can protect nucleic acids through preventing their degradation by nucleases (Klebowski et al., 2018). The unique properties of AuNPs, conjugated to oligonucleotides, can make them potential gene carriers, via covalent and non-covalent bonding. Covalent AuNPs can activate immune-related genes in peripheral blood mononuclear cells, but not in an immortalized and lineage-restricted cell line (Ding et al., 2014). This shows promise application in its application for gene delivery systems. For example, Shahbazi et al. (2019) synthesized AuNPs core using the citrate reduction method, and developed a CRISPR nanoformulation, using colloidal AuNPs (AuNPs/CRISPR), with guide RNA and nuclease on the surface of AuNPs, with or without a single-strand DNA (ss DNA) template to support homology-directed repair. The outcome was an efficient gene editing. They also demonstrated the non-toxicity delivery of entire CRISPR sequences into human blood stem and progenitor cells.

Recently, researchers have also found some evidence that AuNPs can be used as protein carriers. For instance, Joshi et al. (2006) obtained insulin directly bound to bare AuNPs (Au-insulin nanoparticles) via a covalent linkage, which have been confirmed more active than insulin bound via hydrogen bonds with amino acid-modified AuNPs (Au-Asp-insulin nanoparticles) in the transmucosal delivery of drugs for the treatment of diabetes. In this case, the efficiency of insulin delivery can be enhanced by coating the AuNPs with a non-toxic biopolymer, which can strongly adsorb insulin to its surface.

### Therapeutics

**FIGURE 10:**
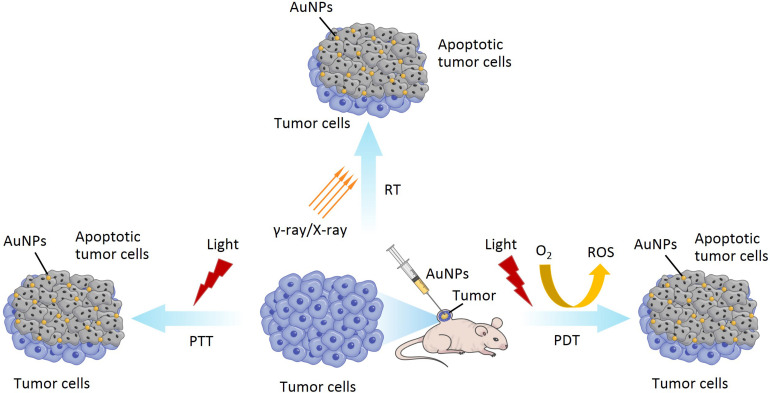
The application of PTT, PDT and RT for AuNPs.

In the following section, we will discuss photothermal therapy (PTT), photodynamic therapy (PDT), and radiation therapy (RT) applications of AuNPs, which continue to be under development (Figure 10).

PTT, also known as thermal ablation or optical hyperthermia, is a non-invasive and is widely applied for cancer therapy due to its benefits of real-time observation of tumor sites and photoinduced destruction of tumor cells or tissues (Singh et al., 2020). PTT uses materials with a high photothermal conversion efficiency, injected into the body, which gather near the tumor tissues by targeting recognition technology (Murphy et al., 2010; Mubarakali et al., 2011). Under the irradiation of external light sources, usually visible or near-infrared (NIR) light, photothermal materials (such as metal nanoparticles) can convert light energy into heat energy (photothermal conversion), result in the destruction of the tumor tissue, and kill the cancer cells (Murphy et al., 2010; Mubarakali et al., 2011). AuNPs as a photothermal material, with maximum absorption in the visible or NIR region, have a high photothermal conversion efficiency due to their SPR effect. In addition, the SPR peak of AuNPs can be adjusted to the NIR region by controlling their geometrical and physical parameters, such as size and shape, which contribute to the depth of effective penetration of PTT (Boyer et al., 2002; Orendorff et al., 2006; Bibikova et al., 2017). Therefore, many researchers have been focusing on the different size and shape of AuNPs for application in PTT (both *in vitro* and *in vivo*) due to their absorption peaks being in the visible or NIR region and their ability to load and deliver various anticancer drugs (Sharifi et al., 2019; Sztandera et al., 2019). AuNPs used in PTT are generally nanorods or nanoshells but, when introduced into a biological environment, the cellular uptake can be limited (Kim and Lee, 2018). Tian et al. (2017) synthesized gold nanostars (AuNSs) with pH (low) insertion peptides (pHLIPs) (AuNSs-pHLIP). They have low toxicity, are plasmon tunable in the NIR region, and exhibited excellent biocompatibility and effective PTT (Tian et al., 2017).

PDT is another form of light therapy, developed in recent decades, and used to destroy cancer cells and pathogenic bacteria (Abrahamse and Hamblin, 2016). PDT involves visible light, photosensitizer (PS), and molecular oxygen (O_2_) from the tissues. PDT is completely dependent on the availability of O_2_ in tissues. The process of PDT is that the PS absorbed by the tissue, is excited by laser light of a specific wavelength. Irradiating the tumor site can activate the PS that selectively accumulate in the tumor tissue, triggering a photochemical reaction to destroy the tumor. The excited PS will transfer energy to the surrounding O_2_ to generate reactive oxygen species (ROS) and increase ROS level in the target sites. ROS can react with adjacent biological macromolecules to produce significant cytotoxicity, cell damage, even death or apoptosis (Imanparast et al., 2018; Falahati et al., 2019; Singh et al., 2020). As a PS, AuNPs can absorb the NIR light, accumulate in the tumor area, raise the temperature, and generate high levels of ROS, which can ultimately damage the tumor growth and promote cancer cell death (Jing et al., 2014). In addition, AuNPs have been considered for PS carriers due to their simple thiolation chemistry for the functionalization of desired molecules, enhancing its capability for loading PS drugs. For example, Yang et al. synthesized spherical AuNPs using UV-assisted reduction with sodium and chloroauric acid, and hollow gold nanorings with a sacrificial galvanic replacement method (Yang et al., 2018). They utilized AuNPs and gold nanorings as drug delivery carriers, with a PS enhancer, to compare and investigate the shape-dependent SPR response in PDT. They found that gold nanorings exhibited efficient PS activation and SPR in the NIR region. Therefore, these may be promising nanoparticles to address the current depth limitation of PDT, for deep tumor therapy.

Besides PTT and PDT, radiation therapy (RT) is one of the least invasive and commonly used methods in the treatment of various cancers (Sztandera et al., 2019). RT involves the delivery of high intensity ionizing radiations (such as γ-rays and X-rays) to tumor tissues, while simultaneously protecting the surrounding healthy cells, tissues, and organs, resulting in the death of tumor cells (Retif et al., 2015; Klebowski et al., 2018). γ-rays and X-rays are usually used to ionize cellular components (such as organelle) and water. Water is the main component of the cell, as well as the main target of the ionizing radiations, resulting in the lysis of the water molecules. This lysis is named radiolysis, which causes the formation of charged species and free radicals. The interaction of free radicals and membrane structure can also cause structural damage, leading to the apoptosis of cell (Kwatra et al., 2013). Recently, there have been many reports of radiosensitization using AuNPs in RT due to their high atomic number of gold (Jain S. et al., 2011; McMahon et al., 2011). The most probable mechanism of radiosensitization from AuNPs is that Auger electron production from the surface of the AuNPs can increase the production of ROS, reduce the total dose of radiation, and increase the dose administrated locally to the tumor sites, eventually resulting in cell death. Moreover, side effects can also be reduced (Jeynes et al., 2014; Retif et al., 2015).

### Diagnostics

**FIGURE 11:**
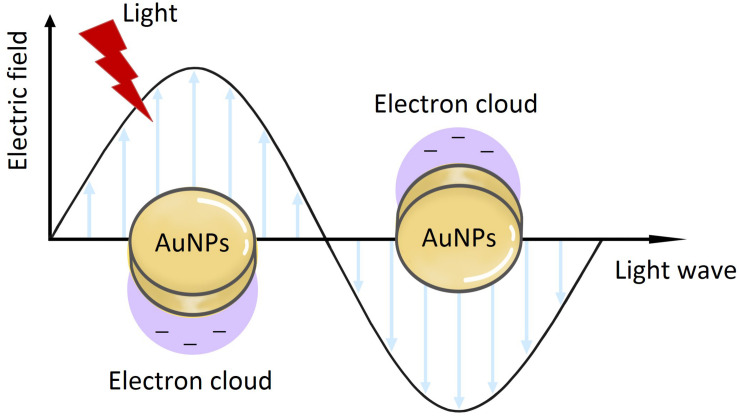
The application of AuNPs on the LSPR.

Diagnostics are very essential to medical science and clinical practice. Some diagnostic methods (such as immunoassay diagnosis) have been applied to clinical diagnosis but have limitations in precision molecular diagnostics because of their inaccuracy and low sensitivity (Ou et al., 2019). With the development of nanotechnology, the sensitivity, specificity, and multiplexing of diagnostic tests have been improved. AuNPs exhibit substantial and excellent optical properties, mainly including localized surface plasmon resonance (LSPR) and surface-enhanced Raman scattering (SERS), which play an important role in their application to diagnostics (Ou et al., 2019; Venditti, 2019). LSPR-based application of AuNPs is due to spectral modulation (Figure 11) (Ou et al., 2019). When the light is incident on the surface of AuNPs, if the incident photon frequency matches the overall vibration frequency of the electrons transmitted by the AuNPs, the AuNPs will strongly absorb the photon energy, and generate LSPR phenomenon, which is useful for diagnostics (Link and El-Sayed, 2003; Liu et al., 2011; Baek et al., 2016; Cordeiro et al., 2016). The LSPR peak of AuNPs is usually in the visible-NIR region, often at around 500 nm or from 800 and 1200 nm (Huang et al., 2009; Aldewachi et al., 2017). SERS is another very attractive spectroscopic technique in diagnostics, being non-invasive and having high sensitivity features (Boisselier and Astruc, 2009; Zhou et al., 2017). Fleischmann et al. (1974) reported the enhancement of a Raman scattering signal, which was the first observation of SERS. The enhancement of SERS can be explained by two mechanisms. One is the chemical enhancement due to charge transfer between gold atoms and molecules (Kawata et al., 2017). Another is the electromagnetic enhancement because of LSPR on the surface of metallic gold (Kawata et al., 2017). Spherical AuNPs are commonly used as the substrate for SERS, although non-spherical AuNPs have also been produced and explored for these applications (Tao et al., 2011; Yang et al., 2012). Nowadays, the phenomenon of LSPR and SERS in AuNPs has been widely used for the development of molecular diagnostics. For instance, El-Husseini et al. (2016) synthesized 15 nm unmodified citrate-coated AuNPs by the Frens method, for use in the diagnostic polymerase chain reaction (PCR) technique for detection of the equine herpes virus 1 (EHV-1). Their results showed that AuNPs-assisted PCR was more sensitive than the conventional PCR technique and, therefore, could be used as a more efficient molecular diagnostic tool for EHV-1.

### Imaging

**FIGURE 12:**
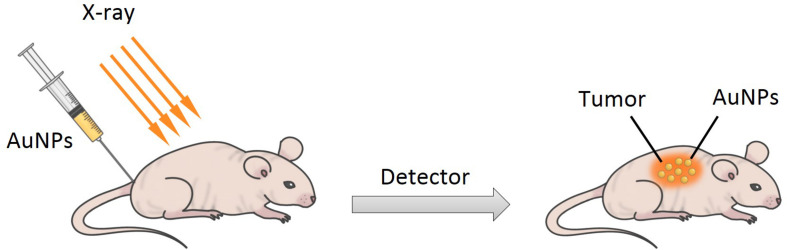
A simple scheme for X-ray imaging.

X-ray computed tomography (CT) is one of the most important and mature tissue imaging techniques widely used in various research and clinical environments with broad availability and fairly low cost (Kim et al., 2007). Specifically, CT is a non-invasive clinical diagnostic tool that can perform 3D visual reconstruction and tissue segmentation (Lusic and Grinstaff, 2013). The images of CT are composed of X-ray images, which are taken at different angles by rotating around an object to form a cross-sectional 3D image called a CT scan (Lusic and Grinstaff, 2013; Fuller and Köper, 2019). According to the content of the images, the contrast agent can attenuate the X-ray to improve the image quality to highlight the specific area, such as the structure of blood vessels or organs (Lusic and Grinstaff, 2013). The basis of CT imaging is the fact that healthy and diseased tissues or cells have different densities, which can generate in a contrast between normal and abnormal cells by using contrasting agents (such as iodinated molecules) (Figure 12) (Cormode et al., 2014). Iodinated molecules are usually used as a contrasting agent, due to their unique X-ray absorption coefficient (Klebowski et al., 2018). However, their usage has its own limitations, such as short imaging times, rapid renal clearance, reduced sensitivity and specificity, toxicity, and vascular permeation (Chien et al., 2012; Mackey et al., 2014). Therefore, it is very essential to explore and develop novel materials as contrasting agents for X-ray imaging. In recent years, AuNPs are attracting attention in imaging as an X-ray contrast agent because they can strongly absorb ionizing radiation to enhance the coefficient of X-ray absorption and convert the light energy to heat energy through the SPR effect (Rahman et al., 2014). Moreover, AuNPs have some advantages compared to iodinated molecules such as ease of synthetic manipulation, unique optical and electrical properties, non-toxicity, higher electron density, higher atomic number of gold, and higher X-ray absorption coefficient (Mackey et al., 2014; Singh et al., 2017). The key factors for potential application of AuNPs in enhanced X-ray CT imaging are their migration and accumulation at target sites and longer vascular retention time, and these allow non-invasive tracking and visualizing of the therapeutic cells (Yin et al., 2017; Meir and Popovtzer, 2018). For example, Liu et al. (2018) synthesized 30–40 nm sized gold nanocages (AuNCs) as part of an activatable probe, to investigate the potential of imaging. The AuNCs were PEGylated *via* conjugation with SH-PEG-NH_2._ It is the first report to estimate protease activity *in vivo* using an imaging technique and activatable probe.

### Others

Besides the various applications described above, some other applications involving antimicrobial (antibacterial and antifungal) activity, antioxidant activity, and anticancer activity need to be mentioned.

The increasing incidence of bacterial infection with drug resistance is a major issue for human health (Dutta et al., 2017). AuNPs are easily taken up by immune cells, due to their excellent cell affinity, which leads to precise delivery at the infected area, facilitating inhibition and damage to microbial pathogens (Saha et al., 2007). AuNPs show excellent antibacterial activity against *E. coli* by absorbing light and converting it into heat (Singh et al., 2009). The growing drug resistance of fungal strains also demands the development of new drugs for better treatment of fungal diseases. Among the various nanoparticles, AuNPs are sensitive to candida cells, which can inhibit the growth and kill the fungal pathogen *C. albicans* (Wani and Ahmad, 2013; Yu et al., 2016). They increase the ROS and damage the cell membrane by their unique properties, which include converting light to heat when irradiated and strong anionic binding with fungal plasma membrane (Wani and Ahmad, 2013; Yu et al., 2016). Cancer is caused by many factors and is considered one of the main causes for death worldwide. In tumor cells, AuNPs have a tendency to enter subcellular organelles and increase the cellular uptake, which enhances anticancer activity (Kajani et al., 2016). AuNPs can increase the ROS level, to destroy cancer cells. However, the biocompatibility and selectivity of AuNPs, in targeting tumors, remains an important challenge. Therefore, new developing methods are required to overcome the question. Excessive ROS can lead to enzyme deactivation and nucleic acid damage, which can itself lead to diseases diabetes, aging, and cancer (Li et al., 2009). Ramalingam (2019) synthesized AuNPs using NaBH_4_ and HAuCl_4_ as a reducing agent and precursor, respectively. Furthermore, they investigated and confirmed the anticancer activity of their AuNPs in human lung cancer cells, and antimicrobial activity against human clinical pathogens, such as *P. aeruginosa*, *S. aureus*, *E. coli*, *V. cholera*, *Salmonella* sp., *K. pneumonia*. Their results suggested that AuNPs could potentially act as anticancer and antimicrobial agents. Moreover, AuNPs have also been confirmed as a potential antioxidant agent. They can inhibit the formation of ROS, thus increasing the antioxidant activity of defensive enzymes. The synergism and antagonism of AuNPs, in their antioxidant activity, require further investigation (Ramalingam, 2019). For instance, Tahir et al. (2015) produced AuNPs (2–10 nm) using the extract of *Nerium oleander* leaf, in a one-step, green synthetic method, and these AuNPs showed good antioxidant activity. Furthermore, the results showed that the extract of *Nerium oleander* leaf was very active for the reduction of AuNPs, and could be used as a reducing agent.

## Conclusion

In summary, since Faraday first reported AuNPs in 1857 (Faraday, 1857), there have been many reports focusing on their synthesis, as well as comparisons with other metallic nanoparticles or noble metallic nanoparticles. In this review, we have described the synthesis and modification of AuNPs, the techniques of characterization, and their diverse medical applications and biological activities. Since the yield is low, using a top–down approach, a series of synthetic approaches to the production of AuNPs have been proposed. Additionally, the unique properties of AuNPs suggest its broad applications, including drug and gene delivery, PTT, photodynamic therapy (PDT), diagnosis, and imaging. Moreover, further applications, arising from their antimicrobial (antibacterial and antifungal), antioxidant, and anticancer activities, have also been discussed. As the properties of AuNPs become better understood, a considerable number of principal experiments and studies are needed to focus on function, along with the design of different therapies, generally involving PTT and PDT. Although the antimicrobial, anticancer, and antioxidant activities of AuNPs have been confirmed, they remain to be used in clinical treatment. As a drug and gene carrier, AuNPs may also have broad applications, in the future. Although AuNPs possess many useful properties, some studies have demonstrated their toxic effects, based on their physicochemical properties. Sabella et al. (2014) showed that the toxicity of AuNPs was related to their cellular internalization pathways. The safety of AuNPs remains a very urgent and controversial issue, as more important concerns are raised, and this needs to be properly addressed. In recent studies, researchers have reduced the toxicity of AuNPs by introducing functional groups to their surface, improved existing methods of synthesis, and have developed new and better methods. In conclusion, the unique properties of AuNPs should be identified, such as their optical properties with SPR bands, and as carriers with anticancer activity, to broaden their applications in various fields.

## Author Contributions

XH wrote the manuscript. YZ collected the literature and generated the figures and tables. TD edited and checked the manuscript format. JL and HZ reviewed the manuscript. All the authors contributed to the article and approved the submitted version.

## Conflict of Interest

The authors declare that the research was conducted in the absence of any commercial or financial relationships that could be construed as a potential conflict of interest.
